# Implementing advance care planning in palliative and end of life care: a scoping review of community nursing perspectives

**DOI:** 10.1186/s12877-024-04888-4

**Published:** 2024-03-28

**Authors:** Katie Wilkin, Mei Lan Fang, Judith Sixsmith

**Affiliations:** 1 NHS Fife, Kirkcaldy, Scotland; 2 School of Health Sciences, University of Dundee, Dundee, Scotland; 3 Urban Studies and Department of Gerontology, Simon Fraser University, Vancouver, Canada

**Keywords:** Advance care planning, Palliative and end of life care, Nurse, Primary care, Education, Confidence, Relationships, Patient, Community nurse

## Abstract

**Background:**

Advance care planninganning (ACP) is a priority within palliative care service provision. Nurses working in the community occupy an opportune role to engage with families and patients in ACP. Carers and family members of palliative patients often find ACP discussions difficult to initiate. However, community nurses caring for palliative patients can encourage these discussions, utilising the rapport and relationships they have already built with patients and families. Despite this potential, implementation barriers and facilitators continue to exist. To date, no research synthesis has captured the challenges community nurses face when implementing ACP, nor the facilitators of community nurse-led ACP. Considering this, the review question of: 'What factors contribute to or hinder ACP discussion for nurses when providing care to palliative patients?’ was explored.

**Method:**

To capture challenges and facilitators, a global qualitative scoping review was undertaken in June 2023. The Arksey and O’Malley framework for scoping reviews guided the review methodology. Six databases were searched identifying 333 records: CINAHL (16), MEDLINE (45), PUBMED (195), EMBASE (30), BJOCN (15), IJOPN (32). After de-duplication and title and abstract screening, 108 records remained. These were downloaded, hand searched (adding 5 articles) and subject to a full read. 98 were rejected, leaving a selected dataset of 15 articles. Data extracted into a data extraction chart were thematically analysed.

**Results:**

Three key themes were generated: ‘Barriers to ACP’, ‘Facilitators of ACP’ and ‘Understanding of professional role and duty’. Key barriers were – lack of confidence, competence, role ambiguity and prognostic uncertainty. Key facilitators concerned the pertinence of the patient-practitioner relationship enabling ACP amongst nurses who had both competence and experience in ACP and/or palliative care (e.g., palliative care training). Lastly, nurses understood ACP to be part of their role, however, met challenges understanding the law surrounding this and its application processes.

**Conclusions:**

This review suggests that community nurses' experience and competence are associated with the effective implementation of ACP with palliative patients. Future research is needed to develop interventions to promote ACP uptake in community settings, enable confidence building for community nurses and support higher standards of palliative care via the implementation of ACP.

**Supplementary Information:**

The online version contains supplementary material available at 10.1186/s12877-024-04888-4.

## Background

'Advance Care Planning' (ACP), also known as 'Anticipatory Care Planning' [1], is a term used in healthcare, describing an informal documented record of a person's life goals, personal values, and/or wishes for future care or medical treatment [1, 2]. ACP is available to adults of any age but is usually initiated with patients in palliative or end-of-life care [2]. The Marie Curie Foundation defines a palliative condition as a person’s life-limiting incurable illness that will eventually lead to death [3]. The World Health Organisation [4] suggests that 56.8 million people require some form of palliative care and service with the most prevalent palliative conditions being Cancer, Heart or Lung disease, Parkinson’s, and Dementia [4, 5].

Patients in palliative care often need to make important decisions about their future care, and the initiation and actioning of Advance Care Plans help to ensure they receive care that is consistent with their life goals, values, and wishes [2, 6]. However, evidence suggests some critique around ACP processes which limits their value for patients [7]. For example, ACP documentation is not always accessible, or when available, clinicians and/or family members may choose not to honour the person’s ACP preferences [7]. As well, unpredicted, or complex care needs at the end of life (i.e., care costs, personal care), may force unforeseen changes to a person’s Advance Care Plan [8].

In addition, Advance Care Plans can be limited in that they are not legally binding. An Advance Care Plan, when transformed into an ‘Advance Directive' (AD) [9] is a legally binding document which expresses patient wishes concerning refusal or acceptance of medical care or treatment if they become incapacitated [10].

While ADs are recognised worldwide, legislation differs across countries [11, 12]. For example, there is legislation in the United Kingdom (UK), such as, the 2005 'Mental Capacity Act’ which enables the appointment of a proxy with Lasting Power of Attorney to make decisions on their behalf, should they become incapacitated [12]. In the United States (US), ‘The Patient Self-Determination Act 1990’ encourages the completion of ADs which legally support patient wishes under State law [13].

While ADs are different from Advance Care Plans in terms of legal standing, both support the person’s treatment wishes in ill-health, palliation, or end-of-life care. However, Advance Care Plans are broader than ADs in that they can identify personal and social wishes and align these to life goals such as place of death (e.g., hospice), housing preferences or desire for resuscitation [14].

ACP uptake varies across countries and regions. In the US, older people, those who are well educated and higher earning tend to complete ACP [15], leaving many younger, less educated, lower-income people without an Advance Care Plan. To increase uptake of ACP, the US Affordable Care Act [16] was introduced to streamline access to health insurance and care costs and thereby reduce health inequalities [17]. According to Knight et al. [18], ACP uptake in the UK is poor. In their national audit of acute hospital admissions in 2020 (covering 123 hospitals) only 4.8% of patients had an Advance Care Plan, despite many of them living with increasing age and illnesses.

As recommended by the UK Royal College of Physicians [19], health professionals caring for people with life-limiting illnesses have a responsibility to initiate ACP discussions. Discussions are best initiated early when a palliative diagnosis has been confirmed, however, this does not always happen, and the creation of an Advance Care Plan will often occur late in the disease trajectory [20, 21]. For Dementia patients, if ACP occurs too late understanding and decisional capacity can be limited, meaning that the ability of the Advance Care Plan to support the person’s autonomy cannot be maintained [22].

Alongside families and carers, nurses are at the forefront of facilitating ACP discussions when caring for patients [6], because they are often the most frequent healthcare contact for patients [23, 24] and tend to have more time with patients than physicians [25]. Having more time enables nurses to generate trust and approach ACP sensitively [26]. In Miller et al., [27] Nurse-led ACP discussions were reported beneficial to palliative patients, yet despite benefits of nurse-led ACP dialogue (e.g., patient satisfaction, built relationships), this communication remains infrequent. As an example, nurses have been known to avoid ACP discussions where they hold time restrictions, low confidence, or little experience in ACP [6, 27–29].

However, patient-nurse communication and/or interactions often present challenges in acute hospital settings, largely due to complex patient care needs [30], busy environments, shortages of staff and/or excessive workloads [31]. As a result, onus of care provision and ACP initiation is often shifted to community care services [32].

Given this, community care settings would appear to offer opportunities for developing Advance Care Plans, especially in the context of palliative care. The UK Department of Health in 2012 [33] reported that palliative patients preferred to be cared for in the comfort of their own home or residential setting when approaching the end of life, placing palliative care with generalist and specialist palliative care professionals (i.e., GP’s, community nurses, specialist nurses). Community nurses may have more opportunities to engage in ACP discussions with their palliative patients, building on topics of death, dying and planning of future care during patient contact time [34]. Specialist palliative care teams usually become involved with patient care at the request of a generalist professional; mainly due to complex symptom burdens [35] or where a patient’s needs exceed generalist resources (e.g., physical, or spiritual care needs) [36].

While previous literature reviews have explored a variety of professional perspectives on ACP, including nurses in both primary and secondary care settings [28, 37, 38], no reviews were entirely focused on community nurses. Consequently, understanding of the role and experiences of community nurses in ACP is limited. The current scoping review addresses this literature gap on community nurse role and experiences of ACP in palliative care. The review aim is to provide a comprehensive understanding of the key factors that shape ACP initiation and implementation within community nursing and palliative care, especially considering the identified conversational and interactional challenges. The review question was: 'What factors contribute to or hinder ACP discussion for nurses when providing care to palliative patients?'. For the purposes of this review, the UK definition of community nursing refers to nurses working in all areas of the community (i.e., care home facilities or people’s own homes) – including district nurses, clinical nurse specialists, community matrons and home nurses [39].

## Methods

A scoping review methodology was used as this type of review is aimed at exploratory mapping of existing knowledge in a research area as well as exposing research controversies and gaps [40]. Scoping reviews are seen as more flexible than other types of reviews [41]. The scoping review was conducted systematically to ensure comprehensive coverage of concepts (community nurses' experience of initiating and implementing ACP), trends (from 2010 to the present) and issues (barriers and facilitators of initiation and implementation). The review was informed by Arksey and O’Malley’s [42] six scoping review stages which are described below in terms of the use of them in this research.

### Stage 1 – Identifying the review question

Review parameters and question development were guided by the Population, Concept, Context (PCC) mnemonic, maintaining a broad scope for literature searching and evidence breadth [43]. Following PCC, the ‘population’ of focus was community nurses, the ‘concept’ was implementation of ACP in palliative care and the ‘context’ was community settings. The review question was: 'What factors contribute to or hinder ACP discussion for nurses when providing care to palliative patients?’.

### Stage 2 – Identifying relevant studies

To answer the review question, a systematic literature search was conducted in June 2021 and updated in June 2023 using the following databases: CINAHL, MEDLINE, EMBASE, and PUBMED. These databases were used as they cover literature in areas relevant to the review question (see Table 1).

**Table 1:** Database searching

Databases, Search Engines and Content-Relevant Websites	*N* = number of articles identified from database searching
*Academic*	
CINAHL	16
Medline	45
PubMed	195
Embase	30
British Journal of Community Nursing	15
International Journal of Palliative Nursing	32
*Total*	333

A combination of search terms was used to identify literature relating to the concepts: 'Advance Care Planning', 'Nursing', 'Primary Care', 'Palliative Care', and 'Perspectives (see Table 2). Search strings and Boolean operators were applied to either narrow or broaden the literature search. In addition, topic-relevant journals were searched for literature available between 2010 to present (The British Journal of Community Nursing and The International Journal of Palliative Nursing): applying the following search terms 'Nurse', 'ACP', 'Advance Care Plan', and 'Community'.

**Table 2:** Search terms used in electronic databases and search engines

Search Terms	
Palliative Care	‘End of Life care,’ ‘Palliative patient,’ ‘Terminal care’ ‘Terminal patient,’
Primary Care	‘Community setting,’ ‘District setting,’ ‘Community-based’
Nurse	‘Nurs*,’ ‘Community Nurs*,’ ‘District Nurse’
Perspective	‘View,’ ‘Perception,’ ‘Experience,’ ‘Feeling,’
Advance Care Plan	‘Anticipatory Care Plan,’ ‘ACP,’ ‘Advance Directive.’
Review	NOT ‘Literature Review,’ ‘Meta-analysis,’ ‘Narrative Review,’ ‘Systematic Review.’

### Stage 3 – Study selection

Eligibility criteria were created using the PCC framework (see Table 3).

**Table 3:** Eligibility criteria*

Inclusion	Exclusion
Published/created between 2010–2023	Published/created before 2010
Qualitative studies or mixed methods with emphasis on qualitative component	Quantitative studies
Studies which were peer-reviewed	Not focused on ACP, end-of-life/palliative care
Available free of charge or are available through university library services	Grey Literature and other studies not peer-reviewed
Focuses on primary care settings or care homes	Require a fee or are not available through university library services
Studies focusing on adults	Secondary care, i.e., hospice, hospital or other
Studies which explored nurse perspectives or experiences with ACP	Studies about paediatrics or young adults
Written/created in the English language	Studies which did not address nurse perspectives or experiences with ACP Resources in languages other than English

^*^Note: no restrictions were made on methodological design

Once database searches had been completed, a de-duplication process was undertaken. Following this, co-author KW screened titles and abstracts using the eligibility criteria. Remaining articles were downloaded and subjected to a full read in a team process involving KW and MF. Hand searching of reference lists identified further articles for a full read. All non-relevant articles were excluded. Any disagreements were resolved by discussion.

**Fig. 1:**
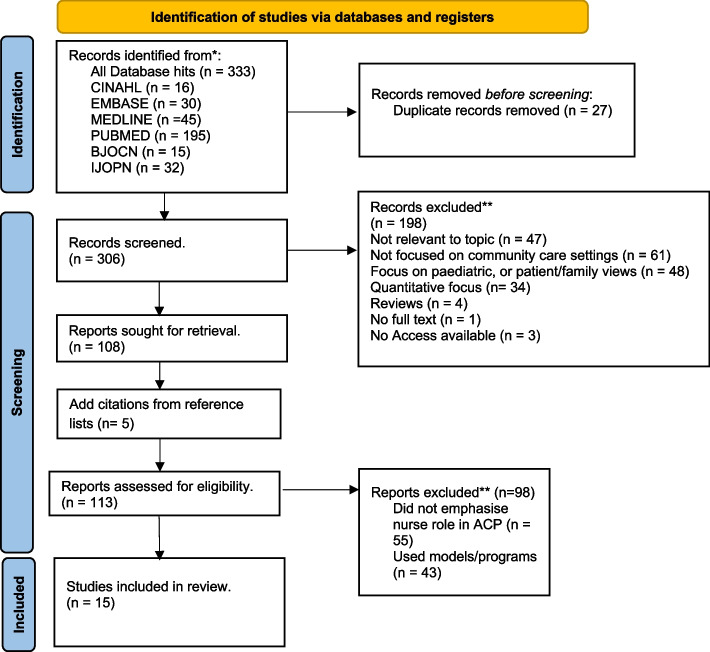
Prisma flow diagram. Legend: The Prisma flow diagram details the systematic search and selection process followed

The article screening process followed 'Preferred Reporting Items for Systematic Reviews and Meta-Analyses Extension for Scoping Reviews' (Prisma-SCr) [44] and the mapping of all database and literature searching results were reported in the Prisma flow diagram [45]. See Fig. 1 for the Prisma flow diagram. For methodological details, refer to Supplementary file 1.

### Stage 4 – Charting the data

A data extraction form was devised to capture the data from selected articles, as suggested by Ghalibaf et al. [46]. Categories of data extraction were: author, year of publication, location, aim/purpose, participants, methodology, type of study, method and key findings. Information identified as relevant was then cut and pasted into the data extraction form under the appropriate category.

### Stage 5 – Collate, summarise, and report the results

To synthesize the data, Braun and Clarke’s [47] 6-step thematic analysis process was applied to data in the extraction form. 1) Familiarisation: Data in the extraction form was read and re-read by the research team before any analysis took place. 2) Qualitative data were coded into meaningful units. 3) Coded data were then amalgamated together based on similarity and identified relationships across the codes. 4) Codes were further combined into ideas for initial themes. 5) The initial themes were discussed by the team and refined to ensure internal consistency and generate final themes. At this point, Bradbury-Jones et al.’s [48] pager framework was used for further analysis. 6) The final written report developed the themes and located them within information gained in a consultancy event (described below). Finally, the key characteristics of the articles (i.e., date of publication) were analysed using descriptive statistics. Table 4 outlines each theme and the articles which align to each theme.

**Table 4:** Articles aligning to each theme

Theme 1 – Understanding of Professional Role and Duty	Seymour Almack and Kennedy 2010 Robinson et al. 2012 Davidson, Bannister and Vries 2013 Menon et al. 2018 Raphael, Waterworth and Gott 2014 Glaudemans et al. 2019	Walshe 2020 Minto and Strickland 2011 Boot and Wilson 2014 Lam et al. 2018 Schichtel et al. 2021 Kastbom, Milberg and Karlsson 2019
Theme 2 – Barriers to ACP Implementation	Seymour Almack and Kennedy 2010 Robinson et al. 2012 Davidson, Bannister and Vries 2013 Menon et al. 2018 Raphael, Waterworth and Gott 2014 Kazmierski and King 2015 Glaudemans et al. 2019	Walshe 2020 Minto and Strickland 2011 Boot and Wilson 2014 Lam et al. 2018 Schichtel et al. 2021 Kastbom, Milberg and Karlsson 2019 Thoresen et al. 2019 Hirakawa et al. 2021
Theme 3 – Facilitators of ACP Implementation	Seymour Almack and Kennedy 2010 Robinson et al. 2012 Davidson, Bannister and Vries 2013 Menon et al. 2018 Raphael, Waterworth and Gott 2014 Kazmierski and King 2015 Glaudemans et al. 2019	Walshe 2020 Minto and Strickland 2011 Boot and Wilson 2014 Lam et al. 2018 Schichtel et al. 2021 Kastbom, Milberg and Karlsson 2019 Thoresen et al. 2019 Hirakawa et al. 2021

The following key themes pertaining to the review aim and question were identified: (1) 'Understanding of professional role and duty', (2) 'Barriers to ACP implementation', and (3) 'Facilitators of ACP implementation'. At this point, a consultation was organised with key knowledge users to gain their perspectives on the review findings.

### Stage 6—Consultation

Consultation offers valuable insight from experts and allows for the identification of any strengths and/or weaknesses in the research [42]. The consultation event was organised as follows: firstly, three experts were identified who worked in academia, nursing, and primary care medicine. A virtual group meeting was organised with the experts using Microsoft Teams. During that meeting, a summary of the review and findings was presented. Next, based on the findings, an open discussion ensued around palliative and end-of-life care in community-based settings. Finally, a set of recommendations for improving palliative and end-of-life care was created. The inclusion of this additional step formulated positive recommendations which in turn, enhanced rigour, and review credibility.

## Results

All articles under review are presented in Table 5. For study characteristics and key findings of all studies see Supplementary file 1.

**Table 5:** Included articles for review

Name	Date	Title	Journal	Volume; Issue	Pages; Doi
Seymour et al	2010	Implementing advance care planning: A qualitative study of community nurses’ views and experiences	BMC Palliative Care	9 (4)	1–9
Minto and Strickland	2011	Anticipating emotion: A qualitative study of advance care planning in the community setting	International Journal of Palliative Nursing	17	278–284
Robinson et al	2012	A qualitative study: Professionals’ experiences of advance care planning in dementia and palliative care, a good idea in theory but	Palliative Medicine	27 (5)	401–408
Davidson et al	2013	Primary healthcare NZ nurses’ experiences of advance directives: Understanding their potential role	Nursing Praxis	29	26–33
Boot and Wilson	2014	Clinical nurse specialists' perspectives on advance care planning conversations	International Journal of Palliative Nursing	20 (1)	9–13
Raphael et al	2014	The role of practice nurses in providing palliative and end-of-life care to older patients with long-term conditions	International Journal of Palliative Nursing	20 (8)	373–379
Kazmierski and King	2015	Role of the community matron in advance care planning and ‘do not attempt CPR’ decision-making: A qualitative study	British Journal of Community Nursing	20 (1)	19–24
Menon et al	2018	Advance care planning in a multicultural family centric community: A qualitative study of health care professionals, patients and caregivers’ perspectives	Journal of Pain and Symptom Management	56 (2)	213–221
Lam et al	2018	Current practices, barriers and enablers for advance care planning among healthcare workers of aged care facilities in western South Wales, Australia	Rural and Remote Health	18 (4)	4714; https://doi.org/10.22605/RRH4717
Thoreson et al	2019	Advance care planning in Norwegian nursing homes – limited awareness of residents’ preferences and values? A qualitative study	BMC Geriatrics	19 (363)	1–6
Kastbom et al	2019	We have no crystal ball – Advance care planning at nursing homes from the perspective of nurses and physicians	Scandinavian Journal of Primary Health Care	37 (2)	191–199
Glaudemans et al	2019	How do Dutch primary care providers overcome barriers to advance care planning with older people? A qualitative study	Family Practice	36 (2)	219–224
Walshe	2020	Aims, actions and advance care planning by district nurses providing palliative care: An ethnographic observational study. British Journal of Community Nursing	British Journal of Community Nursing	25	276–286
Schichtel et al	2021	Implementing advance care planning in heart failure	British Journal of General Practice	71 (708)	e555-e564
Hirakawa et al	2021	Facilitating advance care planning for patients with severe COPD	Home Healthcare Now	39 (2)	81–90

Of the 15 articles, most studies were conducted in the United Kingdom (*n* = 7), with New Zealand (*n* = 2), Australia (*n* = 1), Norway (*n* = 1), Sweden (*n* = 1), Japan (*n* = 1), The Netherlands (*n* = 1), and Singapore (*n* = 1) also represented in this review. The article dates varied between 2010–2023. While no articles were published in 2023, 1 article was published in each of the following years: 2010, 2011, 2012, 2013, 2015, and 2020. 2 articles were published each year in 2014, 2018, and 2021. 2019 was the year in which most articles were published (*n* = 3). In line with the eligibility criteria, all research involved a qualitative or mixed-method approach (looking at qualitative data) which enabled exploration into the experiences of 229 community nurses and the ACP process in practice. The methodological approach of each article varied in terms of the qualitative approach used; phenomenology, ethnographic studies, interpretive and descriptive/research design, action research, exploratory studies, and different forms of analysis such as thematic analysis and latent qualitative analysis.

A range of roles were held amongst community nurses who participated in the studies. These were district nurses/community nurses (*n* = 112) within primary care, specialist community nurses (e.g., Heart failure, COPD) (*n* = 31), nursing home nurses (*n* = 59), community matrons (*n* = 6), and practice nurses (*n* = 21). There was also a variety of community settings, including rural, urban, and suburban settings, multicultural-centric and deprived communities, primary care-providing organisations including community practices or NHS community practice trusts, aged care facilities and nursing homes.

Thematic analysis generated three key themes: (1) 'Understanding of professional role and duty', (2) 'Barriers to ACP implementation', and (3) 'Facilitators of ACP implementation'. All 15 articles identified barriers and facilitators perceived by nurses which influenced avoidance. Only four articles reported findings around the legality of ACP [49–52], with eight articles reporting on community nurse understanding of their role with ACP [49, 50, 52–57]. Further analysis using the pager framework was then conducted to explore patterns, advances, and gaps.

### Theme 1. Understanding of professional role and duty

Four studies found nurses were uncertain about who was legally responsible for leading ACP discussions [49–52]. Two of these studies [49, 50] reported ambiguity about the legislation supporting ACP and Advance Directives (ADs) and formal processes of discussion and documentation of ACP in the UK. This suggests that, in UK contexts, there is a lack of clarity coupled with uncertainty about responsibilities held when communicating or implementing ACP or AD processes. Within the consultation event, discussion covered role ambiguity within UK contexts, suggesting an understanding of the law supporting ACP and ADs needs clarified.

Also, in New Zealand, Davison, Bannister and Vries [51] found that nurses were confused about their own legal responsibility for initiating ACP in practice. Additionally, they did not understand the differences between Advance Care Plans and Advance Directives or how to develop or implement these in practice. Another study, undertaken in Singapore [52], found nurses mirrored similar confusion, not understanding the differences between formal or informal discussions of ACP. They held little understanding of their own role and legal responsibility for implementing ACPs.

Four studies from New Zealand [58], The Netherlands [53] as well as UK [57, 59] indicated that nurses regarded the responsibility of ACP to reside with GPs, who are medically trained. They felt GPs should take the lead in initiating ACP discussions. However, another UK study [54] found that while GP-led ACP was most frequent, some community nurses felt better placed to lead ACP initiation, having more time available for in-depth conversations with their patients; opposed to GP consultations. Boot and Wilson’s UK study [60] suggested that the roles and responsibilities associated with the ACP process should fall to those who know the patient the best, and with whom they already had an established relationship. Yet, six studies found that overall, in this context, responsibility often fell to those with prior experience in ACP (Australia, UK and Singapore) [49, 52, 55, 60, 61].

Conversely, three studies representing Australia [55], the UK [57], and Sweden [56] found that community nurses should lead ACP discussions as opposed to physicians, although the same studies suggested these community nurses mostly initiate the ACP conversation, where physicians then formalise the documentation. In an Australian nursing home, lam et al. [55] found that physicians emphasised efforts to build nurse confidence to initiate ACP conversations, and that having such conversations opened important channels of communication around Advance Care Plans for doctors to then engage with their patients/residents.

### Theme 2. Barriers to ACP implementation

This scoping review identified consistent barriers throughout the literature. All barriers risk hindering nurse engagement in initiating or implementing ACP conversations in practice. The ‘Barriers to ACP implementation’ theme are divided into sub-themes concerning: 1) organisational or system barriers; 2) personal and professional barriers and 3) cultural barriers.

### Organisational or system barriers

Organisational or system barriers are internal barriers that restrict working practices (e.g., access to resources) [62], access to services, or benefits of an organisation for people who use it or work in it [63].

Six studies mostly covering the UK [49, 50, 54, 57, 61], with one Australian study [55], found that a lack of resources presented a barrier to ACP implementation. This was specific to community nurses attempting to balance both expectation of families and patients with the available resources they had for palliative care, in line with a person’s Advance Care Plan. They felt unable to provide optimum palliative care to patients if there were limitations on resources (i.e., time, staffing).

### Personal/Professional barriers

One UK study [57] suggests some nurses who have built strong relationships with patients and families feel introducing conversations about ACP could compromise this relationship; potentially impacting on care. In contrast, from Sweden, Kastbom, Milberg, and Karlsson [56] found that community nurses had trouble communicating about ACP where there was a less intimate relationship with the patient. These authors report that a closer nurse-patient relationship provides ample opportunities for patients to initiate questions about care and end-of-life preferences. Four other studies covering the UK [50, 54, 60] and Singapore [52] exposed nurses to be concerned with how ACP would be perceived by patients and families. Many patients or families remained in denial about a palliative diagnosis or prognosis [52, 60] especially relatives of Dementia patients within nursing homes [50, 64]. Where denial and unrealistic optimism are evident, these authors suggest ACP conversations would bring challenges for nurses.

The disease trajectory and its impact on ACP conversations were mentioned in several articles. Covering the UK [59, 60] and Norway [64] authors reported that the uncertainty of disease trajectories can influence ACP processes, for example, patients may dismiss opportunities for such discussions when well. Conversely, one Japanese study [65] with chronic respiratory patients, reported that patients were more sensitive to ACP, for example, if the topic of ACP was approached during an episode of illness, they felt as if they were receiving a ‘death sentence’ (p.86). Four studies from the UK [54, 60] Sweden [56] and The Netherlands [53] concluded that initiating ACP conversations too early in the disease trajectory had the potential to negatively influence patient reactions and risk distress. As well, the ‘right time’ for ACP conversations was reported difficult to assess [50, 51] and patient and family unreadiness [50, 54, 59, 60], as well as hesitancy [64] towards ACP, was a clear influence on this.

Lastly, a lack of nurse education, knowledge, or competence [50, 57, 58] was another barrier for nurses initiating ACP.

### Cultural barriers

Several studies from the UK [49], Sweden [56], New Zealand [51], and Japan [65], located the ‘curative culture’ as a barrier to ACP (within a broad cultural context). These articles emphasised that medicine focused on a 'curative culture' and, given that palliation is broadly concerned with comfort until death, the ACP process tends to lie outside of curative-focused culture. Community nurse-led ACP discussions are then particularly challenging if unsupported [49, 51, 56, 65]. Curative cultures can impede institutions of best care, for example, in Japan, medical overruling in community settings seen some physicians supporting hospitalisation, despite this in contrast to a patient’s Advance Care Plan.

Complex family dynamics were also identified in terms of cultural barriers to ACP. The cultural context within the family can impact if or when ACP occurs, and family conflicts can make decisions on appropriate support for a relative particularly complicated [54]. Depending on family power structures, the patient's wishes may be dismissed and/or medical opinion overruled [52, 60]. This can result in community nurses avoiding ACP in care situations with family conflict [52, 60].

In terms of the conceptual basis, ACP is conceived of as a difficult and stigmatised topic. As such, the stigma and taboo associated with the topic of ACP was a further barrier seen specific to patients and families [49, 52]. This was often coupled with a general lack of awareness of ACP benefits [49]. As a result, ACP was then perceived by nurses as an uncomfortable topic to broach when patients feared speaking about death or dying [56].

While the literature and consultation point to organisational or system, personal and professional, and cultural barriers, the evidence also indicates a range of facilitators of community nurse initiation of and engagement with ACP.

### Theme 3. Facilitators of ACP implementation

Consistent facilitators of ACP implementation are evidenced throughout the literature. These facilitators assist in bringing about or facilitating something or someone to an outcome such as engagement or communication. The ‘Facilitators of ACP implementation’ theme is divided into similar sub-sections: 1) organisational or system facilitators 2) personal and professional facilitators and 3) cultural facilitators.

### Organisational or system facilitators

UK Community nurses with previous experience felt confident initiating ACP discussions [49, 54, 60, 61]. Two studies from UK [61] and Australian contexts [55] found that when community nurses had additional training from more experienced nurses (i.e., specialist nurses) and observed them regularly implementing and initiating ACP, they then felt better equipped to undertake ACP discussions themselves. Another UK study [54] suggested that learning from other nurses reduced the anxiety around holding such conversations. Structuring ACP communications systematically, in stages, appeared to encourage ACP conversations to happen [61]. In addition, other UK studies [49, 54, 60] as well as an Australian [55] study recognised the importance of preparing for ACP discussions appropriately, with education, experience and/or mentoring. In the consultation event, these preparations were seen as essential for nurse-led ACP. However, both Kazmierski and King [57] and Raphael, Waterworth and Gott [58] (covering the UK and New Zealand) identified that training was not routinely offered to all professionals working with palliative patients, with training offered only to GP’s or district nurses.

### Personal and professional facilitators

All articles, apart from one UK study by Kazmierski and King [57], expressed the potential of good relationships between nurses with patients, families and informal carers facilitating ACP communication [49–56, 58–61, 64, 65]. In addition, ‘building alliances’ [64] and utilising ‘time’ to build relationships and rapport with the relevant stakeholders involved (patients, families, informal carers) were perceived as important mediators of the ACP process [58, 65].

Within Western Countries (UK, New Zealand, Australia), five studies reported patient cues (e.g., patient-initiated conversations about end-of-life) and readiness as key contributing factors for supporting ACP [49, 51, 55, 60, 61]. Additionally, Robinson et al. [50] reported utilising patient ‘cues’ to assess readiness (i.e., a patient or resident openly engages in conversation about their future). In Australia, Lam et al. [55] found where a patient was comfortable speaking about death or dying, this could then be explored. Such ‘cues’ were seen as patient-initiated prompts towards exploring these important conversations about future care [49, 61].

Representing Australia [55], New Zealand [51], The Netherlands [53], UK [54, 61] and Japan [65], six studies highlighted that approaching ACP as a team was an important facilitator. Prioritising multidisciplinary collective efforts towards ACP conversations with patients, streamlined the task and reduced task burden [64], approaching ACP sensitively while alleviating workload pressures [51].

Overall, community nurses shared the perception that ACP was part of good palliative care and enhanced the quality of care they could provide for their patients [51, 56].

### Cultural facilitators

There are two aspects to cultural facilitation identified in the pager analysis: nursing culture, and the patient and family's cultural background. Community nurses work within a culture of nursing care which requires discrete and instrumental care tasks [57] to improve the health and physical comfort of their patients. However, a more incremental engagement with patients, their families and other relevant professionals is often required for effective ACP. For example, from the UK, Walshe [59] stated that nurses might facilitate ACP completion by scheduling home visits to have discussions in stages. This would allow a longer period for patients and families to consider all ACP components. Similarly, in both Norway [64] and the UK [50], building on the topic of ACP in this way was useful for Dementia patients. Nurses in these studies appreciated that this additional time ensured a patient’s cultural sensitivity was maintained (i.e., basic needs, religious needs, or familial beliefs). As well, they felt this planted the seed for patients to consider what matters most to them [56]. Clearly, applying this approach may precipitate patient-initiated ACP communication.

Additionally, the pager analysis enabled the authors to develop implications and recommendations for practice. Table 6 presents the pager analysis.

**Table 6:** Analysis and Interpretation of Study Findings according to PAGER: Patterns, Advances, Gaps, Evidence for Practice and Research Recommendations

*Patterns*	*Advances (Facilitators)*	*Gaps (Barriers)*	*Evidence for Practice*	*Research Recommendations*
*Care and Relationships*	Good strong relationships with patients, residents, families, and colleagues contributed to nurse engagement in ACP Reading patient cues and determining patient readiness was another considerable contributor Nursing culture with focus on improving health and care of patients was evident Respecting the patient’s and family’s cultural background through allowing time with ACP was important	Some of the nurses suggested that ACP had the potential to negatively affect relationships Risk of diminishing hope or causing distress to patients, caused nurses to avoid the topic of ACP Family dynamics/conflicts were identifiable barriers to ACP Some patients and their families did not fully understand ACP or palliative conditions	More home visits where nurses could build on the topic of ACP over several visits could enable and maintain pivotal ACP engagement Sensitive cultural awareness training for nurses would also be beneficial Nurses to have education in ACP and thus, educate their colleagues, patients, residents, and families enabling awareness	Further education and training in ACP for all nurses working in community-based care Cultural training to ensure nurses maintain timely and sensitive ACP Opportunities to enhance communication in difficult topic conversations are recommended
*Workload and Resources*	Engaging ACP from an approach of shared responsibility Optimising this value-based team approach offered continuity and support Adequate resources would enable optimum ACP and end-of-life care or transitions Nurses had more time for ACP than GPs and thought this to be an important facilitator	GPs were known to initiate ACP and prognostic uncertainty had a strong influence The medical hierarchy was a barrier for nurses with ACP as medical focus on ‘curative cultures’ was evident Lack of resources affecting how nurses could deliver optimum palliative care in line with a patient’s ACP was a barrier Increased pressures of workload with restricted resources (i.e., staffing, support)	Appropriate allocation of workload for staff is vital, including the potential placement of designated key workers in care settings for care continuity Readily available resources would ensure wishes of patients can be upheld Auditing and reviewing the workloads as well as resource accessibility for nurses providing palliative care in the community	Evaluating the use of structured tools to support ACP initiation and discussion from community settings as well as the skills required implement these tools in practice effectively Evaluating the effectiveness of shared responsibility of ACP in community nursing settings
*Education and Experience*	Nurses with experience in ACP were more likely to carry out ACP conversations in practice Nurses with formal training in ACP was a facilitator Having a go-to mentor was seen as beneficial to nurses	Nurses without training in ACP or communication were found to disassociate from any ACP task Nurses lacked the skill and confidence to have these conversations in practice	Ensuring there are mentorships or preceptorships available to provide necessary support Ensuring equal opportunities for nurses who would benefit from training in ACP and/or palliative care upskill these nurses in providing ACP for their palliative patients	Provision of Palliative and EOL care training for all nurses who care for these specific patients Mentoring programs for nurses, to enhance confidence and competence in practice should be explored Considering on the job training to open access around training in ACP and/or palliative care

## Discussion

This scoping review aimed to offer a comprehensive understanding of the key factors that shape ACP initiation and implementation within community nursing and palliative care. An analysis of the barriers and facilitators relating to community nurses highlighted several overarching issues which underpin community nurses’ knowledge, perceptions, and role in ACP. These are described below.

### Relationships, communication, and ACPs

Relationships between palliative care providers, patients and their families and rapport arose in the review as strong influencing facilitators of ACP. Such relationships are seen as critical for good palliative and end of life care [65] and provide communication opportunities which support initiating ACP discussions in practice [56]. Head et al. [24] emphasise communicating ACP early with Dementia patients to ensure optimum palliative care. Together, this combination of built relationships, open communication, and patient/family practitioner rapport positively influence ACP discussions by providing these comfortable, social, and relational environments to discuss ACP [66–69].

Aligning with previous research [27, 34], patients value the nurse-led approach to ACP, reporting this approach is more compassionate. As an example, patients feel nurses facilitate deeper considerations towards what matters most to them [27, 34]. However, this review indicated that community nurses could find initiative for ACP conversations difficult. Previous research [22, 34] reports the use of ACP tools for structuring conversations as beneficial. Additionally, these tools enhance patient participation in the decision-making process. However, while used successfully in previous studies [22, 34], it is important to remember that ACP conversations require sensitivity, as well as empathy and enhanced communication skills. Therefore, evaluating these tools to establish how well they embed sensitivity would be important.

The current review identified the value of incremental discussions (i.e., gradually building-on-topics of ACP during each contact time) providing both patient and families time to consider ACP [50, 64]. A possible conflict to topic-building, stated in Östman, Bäck-Petterson, and Sandvik [70], is where the patient prefers continuity as good care and practice suggests. Here, delays may occur regarding ACP initiation if patients are reluctant to speak to nurses they do not know. Nevertheless, topic-building assists developing Advance Care Plans that maintain and preserve the person’s cultural sensitivity [71]; appropriately considering ethnicity, religion, spirituality and/or cultural norms [20], which are all important holistic considerations for ACP [72].

### Patient and/or family readiness

Indicated from this review, community nurses felt assessing the right time for ACP was important [49, 59]. They suggested this assessment can be achieved by evaluating cues and establishing patient and/or family readiness (e.g., open conversations about death and dying). Other studies echoed timing as pertinent for ACP [73–75], although, in this review Walshe [59] reiterates the difficultly in assessing the ‘right time’ when there is uncertain disease trajectories or even stable disease.

In consensus with this review, another literature review by Brooke and Kirk [22] found palliative patients dismissing ACP discussions in stable disease and when feeling well. Dismissing early ACP leads to conversations triggered by ill-health, or deterioration, with little notice for developing care plans at the end of life [17]. As well, this becomes especially problematic if the patient's mental capacity is then compromised [17]. Reassuringly, in this review Thoreson et al. [64] encouraged the reassessment of ACP unwillingness in conditions at risk of mental incapacity such as Dementia. As Harrison Dening’s study [76] reiterates, this would prioritise and maintain the persons autonomy.

Community nurses in this review were reported to avoid initiating ACP conversations in situations of family conflict (i.e., denial) [52, 60] or where family dynamics (i.e., differing priorities) complicated the process [52, 54, 64]. Similarly, in paediatrics, parents and/or carers in denial of a child’s palliative condition presented challenges initiating ACP [77], despite the benefit of ACP preparing families for end-of-life care [78]. However, from this review, Glaudemans et al. [53] mentioned educating families about ACP benefits and building decision-making confidence (e.g., explaining an Advance Care Plan can be amended to reflect changes in care), as well as the confidence of community nurses can help to ameliorate these issues.

### Stigma, palliative care and ACP

In many Eastern and Western societies, stigma continues to be associated with palliative care because of misinterpretations concerning a focus on taboo topics of death and dying [79]. Similarly, ACP can be stigmatised for the same reason. This review highlighted that the stigma around ACP could prevent community nurses as well as patients and their families from engaging in discussions. Overall, a lack of public awareness of the focus of ACP on quality of life as people move towards the end of life and the benefits that accrue from ACP was evident [49]. As Khairuddin et al. [6] and Ng and Wong [80] have already indicated, low levels of awareness influence ACP avoidance.

However, raising public awareness can be difficult and in accordance with Weaver and Vaughn’s [81] 4-year study, is only achievable with consistent ACP education. Hinders [82] addresses that the nurse has a key role in ACP education (e.g., initiating, advocating, and educating patients and families), yet, despite this nursing remit in advocating patient care [81–83], it appears that barriers remain to instituting ACP.

### Resources, team approach, and shared responsibility

In this review, community nurses perceive a lack of resources affected their provision of quality palliative care [49, 50, 54, 57] and as such, how they could provide care in line with a person’s Advance Care Plan. Jimenez et al.’s work [84] also identifies limited resources (e.g., time, staffing), which indicates the healthcare system's poor prioritisation of ACP. Difficulty is faced when professionals (e.g., community nurses) attempt to honour Advance Care Plans without these resources (e.g., support), adding pressure to workloads and responsibilities [85].

Nurses felt that approaching ACP as a team (i.e., sharing the responsibility) helped to alleviate some of the workload associated with the ACP task [51]. Some studies in this review, positioned GPs as better suited to facilitating ACP conversations [56], while others emphasised that multidisciplinary sharing of this responsibility amongst the community nursing team provided more opportunities for initiating and documenting ACPs [51, 53, 61, 65]. Utilising a multidisciplinary approach may therefore be a positive way forward.

While physicians can be often proxied to communicating ACP processes [86], in contrast, this review seen community nurse-led ACP dominant specifically in nursing homes [55, 56]. Chan and Pang [87] reported community nurses already acting as lead ACP facilitators in a similar context. This may relate to the longer stay nature of residents in these facilities, and the impact these relationships have when given adequate time to discuss ACP. However, while some nurses were initiating ACP in their current roles in this review, others remained confused with obvious role ambiguity when it came to formal, informal, and legal requirements of ACP. If community nurses are to stand at the forefront to facilitate ACP, clarity of responsibility should be further addressed. Especially important is to ensure that ACP opportunities are not overlooked because of nurses rationalising the responsibility of ACP to another professional (i.e., GP).

### Education and experience

Community nurses were reported to avoid ACP when they lacked the necessary skills, knowledge, and experience [50, 57, 58]. Brooke and Kirk [22] and Thomas, Lobo, and Detering [88] associated this barrier with nurses having inadequate education or training in palliative care. Reassuringly, findings from Colville and Kennedy [71] indicated that when subject to palliative care training in their study, nurses' awareness of ACP improved as well as their confidence to broach ACP topics in practice.

From this review, community nurses valued having a competent role model. Having this go-to for guidance from an experienced professional (i.e., someone with experience in palliative care) enabled confidence [55, 61]. As mentioned, previous experience or training in palliative care was seen as a contributing facilitator for these community nurses [49, 60, 61]. However, recognisable differences across practices for ACP training was evident. Some practice nurses reported poor access to training because of time constraints [58], where other nurses reported that ACP training would often only be targeted at physicians or towards specific nursing roles (i.e., district nurses) [57, 58]. Nevertheless, Robinson et al. [50] mentioned that some community nurses continued to lack the necessary skills for ACP, regardless of having additional training in this, so considering this when evaluating ACP alongside professional competency is recommended.

### Implications for practice

Several implications for practice were identified in this review. Increasing workloads with inadequate staffing reduces the potential of quality care for palliative patients and poses restrictions on the time nurses may have with their patients to undertake ACP discussions. This review identified that sharing the responsibility of ACP as a team may have the potential benefit of reducing workloads associated with ACP. Conducting workload auditing to establish a review of practice using a team approach would be a positive way forward. The need to reform ACP education and experience is also identified in this review. With evident restrictions on time, exploring on-the-job training, instead of time away from work may improve overall access and opportunities and make use of time more efficiently, so is a further recommendation.

### Strengths and limitations of this study

When presenting early synthesis in the consultation event, experts offered guidance and advice and assisted in identifying patient cohorts and nursing contexts previously omitted from our preliminary findings. The consultation event with expert stakeholders also brought experiential, practice, and academic knowledge together to ensure the relevance of the results to real-world settings and enabled evidence capture which may not have yet reached the academic literature to contribute to the knowledge base.

A scoping review followed a systematic process to make this replicable, transferable, and rigorous ensuring that a comprehensive dataset was identified, and transparent process made evident. This scoping review followed an iterative process, which focused on enhancing literature breadth enabling a flexible approach to capture evidence. A robust review protocol was developed to conduct this review and several tools/frameworks were utilised (i.e., Prisma-Scr, pager Framework, Prisma Flow Diagram) to enhance the overall scoping review process. The tools provided the authors with structured content, optimising quality reporting processes and overall review efficiency (see [42, 44, 45, 48]). An international focus involving eight international articles makes the review findings culturally and internationally transferable.

However, there were limitations in the design and conduction of this review which may have reduced the reliability of the work. In particular, the search strategy was limited to the databases noted in the methods section, and other databases which were not searched could have yielded articles of relevance to the review question. Consequently, how these may have extended or enriched the results is unknown.

As this review was focused on qualitative literature the potential contributions of knowledge gained through quantitative studies are missing, which may have broadened the interpretation and knowledge of factors affecting community nurse delivery of ACP. Search terms were also limited, and the authors acknowledge that other search terms (not included) may have provided a more comprehensive dataset (e.g., future planning, supportive care, long-term care settings, residential care settings and/or nursing homes). In addition, the search was limited to studies published between 2010–2023, and no foreign language literature was included. Such exclusions could have compromised the final dataset.

The process of searching the databases, screening, and study selection was undertaken by one reviewer (co-author: Wilkin) leading to the potential for individual selection bias. McCrae, Blackstock and Purssell [89] suggest that at least two reviewers are needed to reduce the risk of possible selection bias. To mitigate this to some extent, the final subset of articles for full reading was discussed and the final dataset was agreed upon with co-author, Fang.

As well, no quality assessment was undertaken, and if this had been undertaken, this may have enhanced confidence in the veracity of the review conclusions. On sourcing viable literature sources, UK studies dominated and there were obvious limitations met when sourcing studies available from other international contexts, due to restrictions to the English language. Lastly, the review findings were generated from higher income countries, and less is known about how ACP in community nursing is applied in lower income countries.

### Recommendations for future practice

ACP tools or decision aids were identified as a possibility for supporting ACP conversation structure. However, the evidence base regarding the efficacy of ACP tools does not include the communication skills nurses require to use them. This should therefore be further evaluated.

As well, there were no reported findings on ethnicity, spirituality or religious influence and ACP in community settings from these nurses. Without this evidence, it is difficult to determine if further diverse barriers or facilitators exist and further research into this would be recommended.

## Conclusion

Overall, this review has provided a comprehensive understanding of community nurses' perceived experiences of initiating and implementing ACP with their palliative patients. The review question is supported by several identified barriers and facilitators which impact on nurse-led ACP. As well, the nurse’s understanding of professional role and duty with ACP was addressed. In the identification of the renowned similarities of these evidenced facilitators, these can be explored further and ultimately enhance areas for delivery and uptake of ACP.

### Supplementary Information

**Supplementary Material 1.**

## Data Availability

All data generated or analysed during this study are included in this published article (and its supplementary information files).
