# Supplementary material for: Implementing advance care planning in palliative and end of life care: a scoping review of community nursing perspectives
Source: BMC Geriatr. 2024 Mar 28;24:294. doi: 10.1186/s12877-024-04888-4 (PMC10976700; doi:10.1186/s12877-024-04888-4)
Supplement: Supplementary file 1 — Supplementary Material 1. [file 12877_2024_4888_MOESM1_ESM.docx]

**Supplementary File 1**

**Review Protocol.**

**Adapted from Arksey and O’Malley (2005).**

| Stage 1. Identifying the research question. |
| --- |
| Stage 2. Identifying relevant studies. |
| Stage 3. Study selection. |
| Stage 4. Charting the data. |
| Stage 5. Collating, summarising, and reporting the results. |

**Search of Databases**

**CINAHL**

| Search Number | Query | Results |
| --- | --- | --- |
| 1 | Community setting or district setting or primary care or community-based | 164,842 |
| 2 | ‘Healthcare professional’ or nurs* or ‘district nurs*’ or ‘community nurs*’ | 1,001,508 |
| 3 | View or experience or perspective or perception or feeling | 728,688 |
| 4 | Palliative care or terminal care or end of life care or palliative patient or terminal patient | 69,661 |
| 5 | Anticipatory care plan or advance care plan or ACP or advance directive or living will | 10,133 |
| 6 | 1&2&3&4&5 | 32 |
| 7 | 6 & (not) review or ‘literature review’ or ‘systematic review’ or ‘narrative review’ or meta-analysis | 22 |
| 8 | Limit 2010-2021 and English Language | 16 |
| 9. | Add 2021-2023 publications | 16 |

**MEDLINE**

| Search Number | Query | Results |
| --- | --- | --- |
| 1 | Community setting or district setting or primary care or community-based | 317,944 |
| 2 | ‘Healthcare professional’ or nurs* or ‘district nurs*’ or ‘community nurs*’ | 926,799 |
| 3 | View or experience or perspective or perception or feeling | 1,966,015 |
| 4 | Palliative care or terminal care or end of life care or palliative patient or terminal patient | 118,638 |
| 5 | Anticipatory care plan or advance care plan or ACP or advance directive or living will | 22,279 |
| 6 | 1&2&3&4&5 | 63 |
| 7 | 6 & (not) review or ‘literature review’ or ‘systematic review’ or ‘narrative review’ or meta-analysis | 50 |
| 8 | Limit 2010-2021 and English Language | 42 |
| 9 | Add 2021-2023 publications | 45 |

**EMBASE**

| Search Number | Query | Results |
| --- | --- | --- |
| 1 | Community setting or district setting or primary care or community-based | 257,500 |
| 2 | ‘Healthcare professional’ or nurs* or ‘district nurs*’ or ‘community nurs*’ | 813,831 |
| 3 | View or experience or perspective or perception or feeling | 2,040,107 |
| 4 | Palliative care or terminal care or end of life care or palliative patient or terminal patient | 84,919 |
| 5 | Anticipatory care plan or advance care plan or acp or advance directive or living will | 21,776 |
| 6 | 1&2&3&4&5 | 23 |
| 7 | Limit 2010-2021 and English Language | 22 |
| 8 | Add 2021-2023 publications | 30 |

**PUBMED**

| Search Number | Query | Results |
| --- | --- | --- |
| 1. | (community setting or district setting or primary care or community-based) AND ("Healthcare professional*" or nurs* or "district nurs*" or "community nurs*") AND (view or experience or perspective or perception or feeling) AND (palliative care or end of life care or terminal care or terminal patient or palliative patient) AND (anticipatory care plan or advance care plan or ACP or living will or advance directive) NOT (review or "literature review" or meta-analysis or "narrative review" or "systematic review") | 156 |
| 2 | Limit 2010-2021 and English Language | 129 |
| 3. | Add 2021-2023 | 195 |

**British Journal of Community Nursing**

| Search Number | Query | Results |
| --- | --- | --- |
| 1 | Nurse and palliative and acp or advance care plan and community | 10 |
| 2 | Limit 2010-2021 | 10 |
| 3 | Add 2021-2023 | 15 |

**International Journal of Palliative Nursing**

| Search Number | Query | Results |
| --- | --- | --- |
| 1 | Nurse and palliative and acp or advance care plan and community | 27 |
| 2 | Limit 2010-2021 | 27 |
| 3 | Add 2021-2023 | 32 |

**Clarke and Braun's (2013) Six-Step Data Analysis Process:**

Step 1 - Familiarization of data.

Step 2 - Generation of codes.

Step 3 - Combining codes into themes.

Step 4 - Reviewing themes.

Step 5 - Determine the significance of themes.

Step 6 - Reporting of findings.

**Prisma-Scr Checklist**

Preferred Reporting Items for Systematic reviews and Meta-Analyses extension for Scoping Reviews (PRISMA-ScR) Checklist

| **SECTION** | **ITEM** | **PRISMA-ScR CHECKLIST ITEM** | **REPORTED ON PAGE #** |
| --- | --- | --- | --- |
| **TITLE** | | | |
| Title | 1 | Identify the report as a scoping review. | 1 |
| **ABSTRACT** | | | |
| Structured summary | 2 | Provide a structured summary that includes (as applicable): background, objectives, eligibility criteria, sources of evidence, charting methods, results, and conclusions that relate to the review questions and objectives. | 2 |
| **INTRODUCTION** | | | |
| Rationale | 3 | Describe the rationale for the review in the context of what is already known. Explain why the review questions/objectives lend themselves to a scoping review approach. | 3-8 |
| Objectives | 4 | Provide an explicit statement of the questions and objectives being addressed with reference to their key elements (e.g., population or participants, concepts, and context) or other relevant key elements used to conceptualize the review questions and/or objectives. | 7-8 |
| **METHODS** | | | |
| Protocol and registration | 5 | Indicate whether a review protocol exists; state if and where it can be accessed (e.g., a Web address); and if available, provide registration information, including the registration number. | 8 |
| Eligibility criteria | 6 | Specify characteristics of the sources of evidence used as eligibility criteria (e.g., years considered, language, and publication status), and provide a rationale. | 9 |
| Information sources* | 7 | Describe all information sources in the search (e.g., databases with dates of coverage and contact with authors to identify additional sources), as well as the date the most recent search was executed. | 9-10 |
| Search | 8 | Present the full electronic search strategy for at least 1 database, including any limits used, such that it could be repeated. | Supplementary file |
| Selection of sources of evidence† | 9 | State the process for selecting sources of evidence (i.e., screening and eligibility) included in the scoping review. | 10 |
| Data charting process‡ | 10 | Describe the methods of charting data from the included sources of evidence (e.g., calibrated forms or forms that have been tested by the team before their use, and whether data charting was done independently or in duplicate) and any processes for obtaining and confirming data from investigators. | 10-11 |
| Data items | 11 | List and define all variables for which data were sought and any assumptions and simplifications made. | 11 |
| Critical appraisal of individual sources of evidence§ | 12 | If done, provide a rationale for conducting a critical appraisal of included sources of evidence; describe the methods used and how this information was used in any data synthesis (if appropriate). | N/A |
| Synthesis of results | 13 | Describe the methods of handling and summarizing the data that were charted. | 11 |
| **RESULTS** | | | |
| Selection of sources of evidence | 14 | Give numbers of sources of evidence screened, assessed for eligibility, and included in the review, with reasons for exclusions at each stage, ideally using a flow diagram. | Prisma Flow |
| Characteristics of sources of evidence | 15 | For each source of evidence, present characteristics for which data were charted and provide the citations. | 12-13 |
| Critical appraisal within sources of evidence | 16 | If done, present data on critical appraisal of included sources of evidence (see item 12). | N/A |
| Results of individual sources of evidence | 17 | For each included source of evidence, present the relevant data that were charted that relate to the review questions and objectives. | 13 |
| Synthesis of results | 18 | Summarize and/or present the charting results as they relate to the review questions and objectives. | 13-21 |
| **DISCUSSION** | | | |
| Summary of evidence | 19 | Summarize the main results (including an overview of concepts, themes, and types of evidence available), link to the review questions and objectives, and consider the relevance to key groups. | 21 |
| Limitations | 20 | Discuss the limitations of the scoping review process. | 30 |
| Conclusions | 21 | Provide a general interpretation of the results with respect to the review questions and objectives, as well as potential implications and/or next steps. | 32 |
| **FUNDING** | | | |
| Funding | 22 | Describe sources of funding for the included sources of evidence, as well as sources of funding for the scoping review. Describe the role of the funders of the scoping review. | NA |

JBI = Joanna Briggs Institute; PRISMA-ScR = Preferred Reporting Items for Systematic reviews and Meta-Analyses extension for Scoping Reviews.

* Where sources of evidence (see second footnote) are compiled from, such as bibliographic databases, social media platforms, and Web sites.

† A more inclusive/heterogeneous term used to account for the different types of evidence or data sources (e.g., quantitative and/or qualitative research, expert opinion, and policy documents) that may be eligible in a scoping review as opposed to only studies. This is not to be confused with information sources (see first footnote).

‡ The frameworks by Arksey and O’Malley (6) and Levac and colleagues (7) and the JBI guidance (4, 5) refer to the process of data extraction in a scoping review as data charting.

§ The process of systematically examining research evidence to assess its validity, results, and relevance before using it to inform a decision. This term is used for items 12 and 19 instead of "risk of bias" (which is more applicable to systematic reviews of interventions) to include and acknowledge the various sources of evidence that may be used in a scoping review (e.g., quantitative and/or qualitative research, expert opinion, and policy document).

Source: Tricco AC, Lillie E, Zarin W, O'Brien KK, Colquhoun H, Levac D, et al. PRISMA Extension for Scoping Reviews (PRISMAScR): Checklist and Explanation. Ann Intern Med. 2018;169:467–473. [doi: 10.7326/M18-0850](http://annals.org/aim/fullarticle/2700389/prisma-extension-scoping-reviews-prisma-scr-checklist-explanation).

**Data Extraction - Study Characteristics and Key Findings**

| Author/Year | Location | Purpose/Aim | Participants | Methodology | Type of Study | Method | Key findings |
| --- | --- | --- | --- | --- | --- | --- | --- |
| Minto and Strickland (2011) | United Kingdom | Identify the factors that can facilitate or hinder GP and DN ACP engagement. | 3 community Nurses  3 GP’s | Phenomenology | Qualitative | Semi-structured interviews  Face to face | This study identified challenges for both nurses and GPs. There were issues around the emotional labour of ACP and limited resources. There were patients and families who held unrealistic ideas regarding care and ACP. |
| Seymour et al., (2010) | United Kingdom | Determine the nurses understanding of their role in ACP; identify factors which enable engagement or hinder ACP implementation; identify the educational needs of nurses | 23 community nurses | Action Research | Qualitative | Focus groups | ACP was seen to be an important inclusion in nursing practice. Nurses understood advocating for patients was linked to ACP. They highlighted challenges and barriers to carrying out ACP; these included patient/family concerns, time, and resources. They recommended education programs and this education to be incorporated to increase public awareness. |
| Davidson et al., (2013) | New Zealand | Explore senior primary healthcare nurses’ experiences of their role with Advance Directives. | 13 community nurses | Descriptive exploratory research design | Qualitative | Semi-structured interviews | The nurses in this study identified well with their role regarding Advance Directives (AD). The nurses suggested that reflection was key, and that personal experience enabled them to AD. |
| Kazmierski and King (2015) | United Kingdom | Explore the experiences of community matrons with their patients with life-limiting illness and end of life care needs and ACP. | 6 community matrons | Interpretive phenomenological approach | Qualitative | Semi-structured interviews | The nurses within this study identified that while they face many scenarios where discussions involving ACP and DNACPR occur, none of these nurses had received the appropriate education for this. |
| Boot and Wilson (2014) | United Kingdom | Identify the factors and challenges faced, experienced by Clinical Nurse Specialist’s (CNS’s) with terminal care patients. | 12 community-based CNS’s | Phenomenology | Qualitative | Semi-structured interviews | The nurses argued there was a thin line between benefit and harm with ACP. They suggested cues, readiness and physical condition were all enablers in when and if they would instigate ACP to their patients. |
| Raphael, Waterworth and Gott (2014) | New Zealand | Explore the practice nurse role in caring for patient with long term conditions and end-of-life care needs. | 21 practice nurses | Qualitative descriptive study | Qualitative | Semi-structured interviews by telephone. | End of life care was emphasised a key role for these practice nurses. The nurses were perfectly placed to discuss ACP with their patients, however, many argued to hold little involvement in these processes. |
| Menon et al., (2018) | Singapore | Explore the attitudes and perspectives of health professionals, patients, and caregivers regarding ACP in a multi-centric community. | 15 doctors  13 nurses  5 medical social workers  15 patients  13 caregivers | Exploratory  Study | Qualitative | In-depth interview  Focus Groups | There was confusion regarding legal capacity and ACP. It was identified that many participants felt ACP did not offer value as families and family decision-making held the overpowering position. |
| Schichtel et al., (2021) | United Kingdom | To determine the perspectives of primary care practitioners and how to promote ACP engagement in HF patients. | 17 GP’s  7 community nurses | Interpretive and descriptive | Qualitative | Semi-structured interviews  Face to face | Many of the healthcare professionals were challenged with ACP, where they held personal fears, concerns of prognostic uncertainty and concerns of patient readiness for ACP. They considered ways to overcome these challenges, promoting shared teamwork and educating staff in disease-specific training. |
| Hirakawa et al., (2021) | Japan | Explore and determine the factors associated with healthcare professionals and implementing ACP with adult COPD patient in the community. | 6 physicians  19 nurses  4 social workers  1 care manager | N/R | Qualitative | Semi-Structured interviews  Focus Groups | The healthcare professionals identified several challenges to ACP, these focused on legality and ethics, communication skills and information and understanding of ACP amongst a team of professionals. |
| Glaudemans et al., (2019) | Netherlands | To examine how these primary care providers experience overcoming ACP challenges/barriers with older people. | 8 GP’s  3 nurses  3 elderly care physicians | N/R | Qualitative | Interviews | The nurses were evidenced as reflective practitioners and actively pursued education around ACP. They met challenges where the patient and family’s eagerness and openness to ACP were low and investigated ways in which they could overcome this. |
| Kastbom, Milberg and Karlsson, (2019) | Sweden | To investigate clinicians’ perspectives on the factors that shape the process of ACP in a nursing home context. | 14 physicians  11 nurses | Latent qualitative analysis | Qualitative | Interviews | The clinicians in this study categorised ACP to explore end of life wishes, incorporate patient and families, include decisional documentation, and can review and disregard ACP if they felt this was necessary. |
| Lam et al., (2018) | Australia | To provide an overview of perspectives of practice and attitudes of ACP and AD’s in Care facilities in Australia. | 46 nursing assistants  37 nurses  1 OT  4 therapists  4 doctors  13 others | Cross-sectional survey | Mixed methods | Survey, questionnaire,  Semi-structured interviews | The study highlighted that training was regarded from (81%) of participants as beneficial, increasing engagement in ACP. There were identifiable barriers including capacity and staffing, although these were overcome where there were supportive communities. |
| Thoresen et al., (2019) | Norway | To explore the healthcare professionals’ experiences with ACP in nursing homes. | 11 nurses  8 physicians  1 nursing assistant  1 nursing student | N/R | Qualitative | Semi-structured group interviews | This study evidenced that there was an importance in balancing the benefits and risks of ACP. There was recognised conflict from families, dominating residents’ choice decisions. |
| Walshe (2020) | United Kingdom | To examine the district nurses’ role in palliative care and explore this in practice. | 11 district nurses | Ethnographic Study | Qualitative | Non-participant observation, in-depth interviews | ACP was emphasised in this study as proxy to future care planning. Nurses in this study were observed caring for their patients, where they demonstrated strong relationships with patients and families. |
| Robinson et al., (2012) | United Kingdom | To critically examine the perceptions of various healthcare professionals on ACP. | 5 specialist palliative care professionals (consultant and CNS)  10 GP’s  39 nurses  6 Ambulance workers  3 solicitors  7 Volunteers | N/R | Qualitative | Focus groups  Semi-structured interviews | This study identified uncertainty over the need for ACP. There was evidence of role confusion and responsibility of ACP­­­­­­. Timing was also a hinderance to these professionals when caring for dementia patients. |

***Note N/R – indicates not reported.**

| **Journal Article and key findings aligning with the themes of this review.** | **Theme 1 - Key Identified Barriers** | **Theme 2 - Key Identified Facilitators** | **Theme 3 - Understanding of Nurse Role and Responsibility with ACP** |
| --- | --- | --- | --- |
| Seymour Almack and Kennedy 2010 | - Nurses lacked confidence in understanding all components of ACP. - Care often focused on curative cultures. - Lack of resources to meet expectations and needs of patients with ACP, in end-of-life care situations. - Lack of patient or public awareness of ACP and managing expectations of end of life. - Fear of death and dying, taboo subject. - Managing patient, carer, and family conflicts. - Prognostic uncertainty. - Time consuming - Unpredictable workloads. | - Patient cues, and patient-initiated prompts towards discussions. - Building relationships with families of patients, to improve communications and identify issues using ACP. - Nurses seen documenting ACP, patient wishes, and values-enabled advocacy. - Enabling ACP supported by more experienced colleagues who had training or experience in implementing this. | - Lack of understanding of the nurse’s role with ACP, and legislation supporting this. - Understood they had a role in ‘opening the door’ towards ACP discussions with their patients. |
| Robinson et al 2012 | - Difficult topic to broach - Lack of skills to successfully implement ACP with or without training. - Unclear of when is the ‘right time’ to have these discussions with some patients (e.g., dementia). - Prognostic uncertainty | - Using cues to assess patient readiness, talking about the future. | - Uncertainty around whose role and responsibility was ACP in terms of the more legal and formal discussions. - Community nurses felt ACP was a responsibility of their role. |
| Davidson, Bannister and Vries 2013 | - Difficult or sensitive subject. - Curative / medically focused culture. - Overruling from medical opinions. - Assessing the ‘right time’ to discuss ACP. | - Recognised this to be advocating for the patient. - Trust and rapport with patients and families. - Collaborated team approach to support ACP with patients. - Building on the topic of ACP with time. | - Primary care nurses held a lesser role in implementing ACP or Advance Directives. - All nurses were in favour of ACP/Advance Directives. - Confusion around the legality of ACP/Advance Directives. - Confusion about how to implement or develop ACP/Advance Directives. |
| Menon et al 2018 | - Patient unreadiness - May cause loss of hope, or depression/distress to patients and their families. - Taboo subject | - Previous experience in ACP - Nurses holding good relationships with patients and families | - Confusion was evident about the legal framework of ACP, where many misunderstood their own role and the legality of their role in relation to this. - Some nurses lacked awareness of ACP and confused this with ADs. |
| Raphael, Waterworth and Gott 2014 | - Inability to carry out home visits with patients. - Lack to time to seek further training or education in palliative care. - Training often targeted at GP’s not nurses. - Trying to define when the condition becomes terminal. - Mixed input from generalist and specialist palliative care services. | - Formal education in palliative care. - Building relationships with patients and families. - Time to build rapport. | - Limitations, as roles were often GP-led when dealing with end-of-life care and ACP discussions. |
| Kazmierski and King 2015 | - Unpredictability of illness and disease trajectories in long-term conditions. - No formal training in ACP/DNACPR discussions. - Training offered to district nurses and not necessarily community matrons. - ACP is perceived as negative by the patient and their family. | - Knowing their patient and building a relationship. - Collaborative working with district nurses, Macmillan nurses and specialist nurses to support palliative patient’s wishes. | - Nurses would initiate but not formalise ACP as this would be formalised by a medical professional. |
| Glaudemans et al 2019 | - Fear of causing loss of hope or distress. - Lack of time to discuss ACP sensitively. | - Good communication skills. - Spending time, preparing patients and families for steps of ACP. - Home visits by nurses providing information to patients and families in smaller portions, building on the topic of ACP. - Building rapport and relationships with patients and families. - Collaborated MDT approach, nurses worked with GPs towards ACP. | - Believed that conversations can be initiated by nurses, however, nurses felt the full responsibility fell to the GP to make decisions and document ACP with patients and families formally. |
| Walshe 2020 | - Patients dismissing the topic of ACP/unreadiness. - Uncertainty of patient disease, illness and/or prognosis. | - Built relationships with patients and families. - Patient initiated discussions towards ACP. - Patient cues - Planning and preparing for home visits; time for discussions. | - GPs were known to lead ACP discussions. |
| Minto and Strickland 2011 | - Concern of causing patient distress. - Increasing workloads and constraints, being unable to meet end-of-life care expectations. - Limited resources to meet patient and family expectations. - Uncooperative or unsupportive family and dynamics. | - Previous experience builds confidence in ACP. - Learning from others, reduces professional anxieties. - Good communication with family, and patient relationships. - Shared responsibility and close working relationships. | - Recognised as a shared responsibility when caring for palliative or end-of-life patients. |
| Boot and Wilson 2014 | - Patient unreadiness - Disjointed family dynamics - Uncertainty of disease trajectory - Difficult, or uncomfortable topic. | - Patient Readiness - Patient/family/nurse relationship - Experience in implementing ACPs | - Nurses felt ACP role and responsibility should fall to the person who held a relationship with the patient. |
| Lam et al 2018 | - Little or no training in ACP available. - Working often with limited resources in the residential care settings. - When patients lacked the mental capacity for decision-making. - Unsupportive family | - When comfortable speaking about death and dying. - Time was a facilitator. - Good relationship with the patient and their family. - Cooperative families. - Experience in ACP | - Seen ACP was the responsibility of everyone in the residential care settings. - Did not understand sole responsibility fell to the GP/doctor. - Nurses frequently initiated ACP conversations in their role. |
| Schichtel et al 2021 | - Fear of death and dying and nurses being uncomfortable about these conversations. - Nurses felt they Lacked the skills for speaking about ACP. - Lack of time was a barrier. | - Further training in end-of-life care especially disease specific end of life care. - Using a template guide towards conversations in ACP. - Question prompts from the patient, obvious readiness. - Improved communications from specialists with primary care. | - Some felt the specialised and experienced specialists should initiate these conversations or acknowledge the requirement of this to the primary care team to then explore. |
| Kastbom, Milberg and Karlsson 2019 | - Not knowing the patient or having a relationship with them was a barrier. - Communication and documentation issues with primary care and nursing homes. - Fear of speaking about death and dying with patients. - Prognostic uncertainty. | - Nurse and patient/family relationship. - Continuity of care, building relationships and getting to know patients. - Preparing patients and families before any discussion, planting the seed for them to start considering ACP. | - Nurses would opt to leave ACP or such discussions with medical leads when they could not provide continuity or where they held no relationship with the patient. - Understood they could collaborate discussions by preparing the patients to have these discussions with their GP. |
| Thoresen et al 2019 | - Family or patient denial of actual illness. - Disjoint in family dynamics, and opinions. - ACP coupled with stress, difficulty, and time consuming. - Residents' or families’ preferences are unclear or hesitant. - Little to no training in ACP implementation or difficult conversations. | - Built alliances with families of residents. - Collaborated MDT approach may improve implementation. | - Not reported. |
| Hirakawa et al 2021 | - Patients may feel they are receiving a death sentence if a nurse was to discuss ACP at the wrong time. - Lack of time to discuss the aspects of ACP with patients. - Unpredictability of the COPD illness. - Medical led inappropriate admissions to hospital, hierarchy avoiding ACP discussions. | - Sharing the responsibility of ACP as a team. - Good relationships built over time with patients and their families. | Not reported. |
